# Editorial: Beyond genetics: modifications of nucleic acid and chromatin

**DOI:** 10.3389/fpls.2023.1223443

**Published:** 2023-05-31

**Authors:** Jungnam Cho, Daniel Schubert, Yue Zhou

**Affiliations:** ^1^ National Key Laboratory of Plant Molecular Genetics, CAS Center for Excellence in Molecular Plant Sciences, Shanghai Institute of Plant Physiology and Ecology, Chinese Academy of Sciences, Shanghai, China; ^2^ Epigenetics of Plants, Freie Universität Berlin, Berlin, Germany; ^3^ State Key Laboratory of Protein and Plant Gene Research, School of Advanced Agricultural Sciences, Peking-Tsinghua Center for Life Sciences, Peking University, Beijing, China

**Keywords:** epigenetics, auxin biosynthesis, cell pluripotency, floral transition, polymorphisms

This Research Topic “*Beyond genetics: modifications of nucleic acid and chromatin*” gathers different contributions highlighting epigenetic modifications involved in diverse aspects of fundamental biological processes in plants. These studies shed light on the mechanisms as to how epigenetic modifications control auxin biosynthesis, cell pluripotency, floral transition, and DNA polymorphisms in model and non-model plant species.

The first article of this Research Topic (Wang et al.) summarizes the epigenetic regulatory mechanisms of auxin biosynthesis. Indole-3-acetic acid (IAA), the major natural auxin, is synthesized through tryptophan-dependent and -independent pathways. The authors introduce that histone modifications, chromatin remodeling, DNA methylation and small RNAs all participate in regulation of auxin biosynthesis genes. *YUCCA* (*YUC*) (Cao et al., 2019) family genes, encoding the enzymes that catalyze the conversion of indole-3-pyruvic acid to IAA, are the main targets for histone modification. The chromatin remodeling complex can also regulate *YUC* gene expression by controlling the replacement between canonical histone H2A and variant histone H2A.Z. Small RNAs are usually coupled with DNA methylation through the RNA-directed DNA methylation (RdDM) pathway to regulate auxin biosynthetic genes, while DNA methylation is involved in tissue-specific gene expression. Epigenetic modifications integrate responses to changes in the abiotic environment, such as shade avoidance and sensing ambient temperature, and auxin biosynthesis. The review points out that further investigations are needed to elucidate the epigenetic regulation of other auxin biosynthesis genes, regulation of tissue-specific expression patterns by various histone modifications, and the cross-talk and coordination of multiple epigenetic modifications.

The second article of this Research Topic (Muller-Xing and Xing) shows the relationship between epigenetic modifications and cell pluripotency. The authors suggest that the epigenetic mechanisms play a crucial role in the initiation, maintenance, and determination of plant stem cells. Epigenetic factors control stem-cell fate by regulating either pluripotency genes directly or genes that maintain meristem organization. In shoot and root apical meristem (SAM and RAM), the pluripotency genes are controlled by histone variants deposition, histone modifications associated with gene activation or repression and chromatin remodeling complexes. Although chromatin modifiers are unable to bind DNA directly, many studies have found that intermediary transcription factors, pluripotency factors and cell cycle regulators can connect epigenetic factors and target DNA. Histone modifications are also involved in *de novo* stem cell formation through suppressing the pluripotency genes in differentiated tissue. Similarly, epigenetic silencing of several pluripotency genes also leads to stem cell determinacy in flower development. *De novo* stem cell regeneration contributes to organogenesis and relies on the removal of repressive H3K27me3 at pluripotency genes. It is still to be discovered how the epigenetic pathways corporate with each other and which upstream signals modulate their spatiotemporal specificity.

Further to the question as to how epigenetic modifications regulate developmental decisions, Lim et al. show that the HOS15-PWR-HDA9 complex negatively regulates the floral transition in Arabidopsis. This study suggests that the floral activator *AGL19* is activated by the histone deacetylation mediated by the HOS15-PWR-HDA9 complex. By applying RNA-seq, the authors found that, similar with the previously reported repressive function of the PWR-HDA9 complex, HOS15 might repress the *AGL19* expression, concordantly with its early flowering mutant phenotype. The results of ChIP-qPCR revealed that together with the PWR-HDA9 complex, HOS15 also binds to the promoter regions of *AGL19*. This work complements our current knowledge of epigenomic control of flowering time.

An improvement in studying chromatin states is a timely and concerning issue, thus a novel and optimized approach based on Fluorescence activated nuclei sorting (FANS) is proposed in Wang et al. To overcome the technical barriers of cell walls and chloroplasts in mapping cis-regulatory elements and exploring transcription regulation, Wang et al. developed a new experimental and data analysis method to map open chromatin in wheat. In the optimized method, chopping the tissue instead of grinding it in liquid nitrogen is suitable for small amount of tissues. A buffer with 0.4 M concentration of D-Sorbitol can achieve effective nuclei extraction and Tn5 tagmentation in various wheat tissues. The nuclei were stained with 4, 6-Diamidino-2phenylindole (DAPI) and then subjected to Fluorescence activated nuclei sorting (FANS). In the data analysis pipeline, strict alignment conditions and reproducibility checks are used to suit wheat and other plant species. This method requires fewer number of cells and robustly performs in diverse tissues, thus providing a new and versatile approach to epigenetic research field in wheat and potentially to other plant species as well.

In addition to histone modifications, another study focuses on nuclear DNA diversity (Yu et al.). In this article, the authors describe the intraorganismal genetic heterogeneity (IGH) in *Leymus chinensis*. The authors first detected that in *Leymus chinensis* leaves, the nuclear genes (*VIPP1* and *XLG3*) had higher nucleic acid polymorphism compared to the chloroplast genes (*psbA* and *ndhH*) by sanger sequencing. This higher nucleic acid polymorphism was further validated by high-throughput sequencing and the observation of normal cell size. By calculating the mutation frequencies, it was found that the mutation rates were predisposed. Next, the authors detected polymorphisms which started to accumulate from the seed stage and were subsequently reduced during development, meanwhile polymorphisms detected in leaves rarely resulted in amino acid changes, while they did in seeds. These observations in *Leymus chinensis* represent an exception to the pattern of accumulation of somatic mutations resulting in potential deleterious effects, and *Leymus chinensis* clearly shows strong ‘purifying selection’ to constrain the variation at the protein level.

In summary, this Research Topic covers various aspects of epigenetic regulation in auxin biosynthesis, cell pluripotency, floral transition and polymorphisms, from discoveries to method development. All these studies will undoubtedly promote further progress in this field. We hope that the reader will find this Research Topic a useful reference for the state of the art in the emerging field of epigenetics and cutting-edge methods. Moreover, we thank all authors and reviewers who contributed to this Research Topic.

## Author contributions

All authors listed have made a substantial, direct, and intellectual contribution to the work and approved it for publication.
